# A computational study of the effects of size, location, and number of tears on haemodynamics in surgically repaired type A aortic dissection

**DOI:** 10.3389/fcvm.2023.1215720

**Published:** 2023-06-14

**Authors:** Kyosuke Motoki, Yu Zhu, Saeed Mirsadraee, Ulrich Rosendahl, John Pepper, Xiao Yun Xu

**Affiliations:** ^1^ Department of Chemical Engineering, Imperial College London, London, United Kingdom; ^2^ National Heart and Lung Institute, Imperial College London, London, United Kingdom; ^3^ Department of Radiology, Royal Brompton and Harefield Hospitals, London, United Kingdom; ^4^ Department of Cardiac Surgery, Royal Brompton and Harefield Hospitals, London, United Kingdom

**Keywords:** type A aortic dissection, computational fluid dynamics, haemodynamics, re-entry tear, aortic dilatation

## Abstract

**Objective:**

This study aimed to comprehensively examine the roles of size, location, and number of tears in the progression of surgically repaired type A aortic dissection (TAAD) by assessing haemodynamic changes through patient-specific computational fluid dynamic (CFD) simulations.

**Methods:**

Two patient-specific TAAD geometries with replaced ascending aorta were reconstructed based upon computed 15 tomography (CT) scans, after which 10 hypothetical models (5 per patient) with different tear configurations were artificially created. CFD simulations were performed on all the models under physiologically realistic boundary conditions.

**Results:**

Our simulation results showed that increasing either the size or number of the re-entry tears reduced the luminal pressure difference (LPD) and maximum time-averaged wall shear stress (TAWSS), as well as areas exposed to abnormally high or low TAWSS values. Models with a large re-entry tear outperformed the others by reducing the maximum LPD by 1.88 mmHg and 7.39 mmHg, for patients 1 and 2, respectively. Moreover, proximally located re-entry tears in the descending aorta were more effective at reducing LPD than distal re-entry tears.

**Discussion:**

These computational results indicate that the presence of a relatively large re-entry tear in the proximal descending aorta might help stabilize post-surgery aortic growth. This finding has important implications for the management and risk stratification of surgically repaired TAAD patients. Nevertheless, further validation in a large patient cohort is needed.

## 1. Introduction

Aortic dissection (AD) starts with a tear in the intima of the aortic wall, which allows blood to enter the medial layer, developing a secondary channel of blood flow, known as “false lumen” (FL) alongside the original “true lumen” (TL). According to the Stanford classification system, dissections arising from the ascending aorta and the arch are called type A aortic dissection (TAAD), while those confined to the descending aorta are called type B aortic dissection (TBAD).

The standard treatment method for acute TAAD is open heart surgery with cardiopulmonary bypass, which involves replacing the dissected aortic segment with a synthetic graft. The hospital mortality in UK ranges from 10% to 22%. However, a late mortality rate of 29.3% was reported as a result of aortic rupture (1), which might be attributed to unresected re-entry tears in the distal regions due to procedural complexity and significant risk involvement (2). There is currently a limited understanding of the key distinguishing factors between patients with a high risk of developing progressive aortic dilatation (PAD) following TAAD surgical repair and those without.

Extensive studies of anatomical influences on dissection-related adverse events and mortality have been reported for TBAD. Specifically, the number of re-entry tears, as well as the size and location of entry tear have been associated with late complications (3–6). Substantial efforts have been made to study the underlying mechanisms and some studies have reported that occlusion of distal tears could lead to significantly elevated FL pressure, which would further result in FL dilatation (7, 8). On the other hand, increasing the number of re-entry tears was reported to reduce FL pressure (9–13), thereby equalizing TL/FL luminal pressure difference (LPD) (9–11, 13–15). Additionally, larger entry tear leading to higher FL flow (16) was found to cause progressive aortic dilatation (17). Despite showing insightful information on the roles of tears in haemodynamic changes, these studies were limited to TBAD.

Haemodynamic characteristics in TBAD are different from those in repaired TAAD patients, with the latter presenting with higher TL and FL kinetic energies and lower FL stasis (18). In our previous study, significantly higher LPD and fewer number of re-entry tears were observed for repaired TAAD patients with unstable aortic growth (>2.9 mm/year) (19). This observation confirmed a potential role of re-entry tears in pressure stabilisation and called for further investigations regarding anatomical influences on haemodynamics in repaired TAAD patients.

Therefore, this study aimed to comprehensively examine the impact of size, location, and number of re-entry tears, on haemodynamics in surgically repaired TAAD patients. Two repaired TAAD patients were included, whose geometries were reconstructed from patient-specific computed tomography angiography (CTA) images. Based on the reconstructed patient-specific geometries, hypothetical models with different re-entry tear configurations were created. Computational fluid dynamics (CFD) simulations were then carried out, and the predicted haemodynamic parameters including flow patterns, TAWSS and LPD were compared between all the simulated models.

## 2. Methodology

### 2.1. Data acquisition and geometry reconstruction

This study was approved by the institutional committee of the Health Research Authority (HRA) and Health and Care Research Wales (HCRW) on May 4, 2020 (ref: 20/WM/0145), and the need for informed patients' consent was waived.

Two patients with repaired TAAD were retrospectively selected from the validated database at the Royal Brompton Hospital. Patient 1 was a 39-year-old male who underwent a Bentall procedure with aortic valve replacement for TAAD. Patient 2 was a 74-year-old male who had emergency replacement of the aortic root and ascending aorta for TAAD. The CTA images used in this study were obtained 10 months and 13 months after the aforementioned operations, for patient 1 and 2, respectively. Comparing to a later CTA scan showed a progressive aortic dilatation (>2.9 mm/year) in the residual dissected descending aortas of both patients.

Both patients were examined through a SOMATOM Definition Flash CT scanner (Siemens Healthineers), and the images were reconstructed with 0.75-mm slice thickness and 0.5-mm slice increment. Patient-specific geometries were reconstructed from the CTA data using an image analysis software Mimics 24.0 (Materialise, Belgium). Briefly, 2D masks containing the regions of interest were manually segmented based on local greyscale intensities, which were then integrated to generate a 3D fluid domain, followed by 3D surface smoothing. The computational model was created from the aortic sinotubular junction to the level just proximal to the iliac bifurcation, with three main arch vessels being included. For each patient, five additional hypothetical models with different tear configurations were artificially created, in order to examine their effects on local flow.

Anatomical features including the tear size and distance were measured and summarized in Table 1. Patient 1 presented with a primary entry tear located distal to the left subclavian artery (LSA), together with six re-entry tears: two in the proximal descending aorta (DA) and four in the distal region. Patient 2 presented with a primary entry tear in the proximal aortic arch and two re-entry tears: one in the proximal arch and another in the distal abdominal aorta. It should be noted that the “primary entry tear” referred to in this study is the most proximal tear in the residual dissected aorta whereas the original primary tear in the ascending aorta was resected during surgery. The re-entry tears were defined as communications between the TL and FL, located distally to the primary entry tear.

**Table 1:** Summary of measured area of each tear and distance between each tear and LSA. .

	Patient 1						
	Entry tear	Re-entry tear number					
		1	2	3	4	5	6
Area (mm^2^)	108.4	10.6	3.5	16.1	6.6	1.6	2.1
Distance between each tear and LSA (mm)	36.3	57.7	135.1	270.7	319.4	324.7	344.5
	Patient 2						
	Entry tear	Re-entry tear number					
		1			2		
Area (mm^2^)	10.7	84.1			88.7		
Distance between each tear and LSA (mm)	−38.8	−5.7			348.5		

LSA, left subclavian artery.

**Figure 1:**
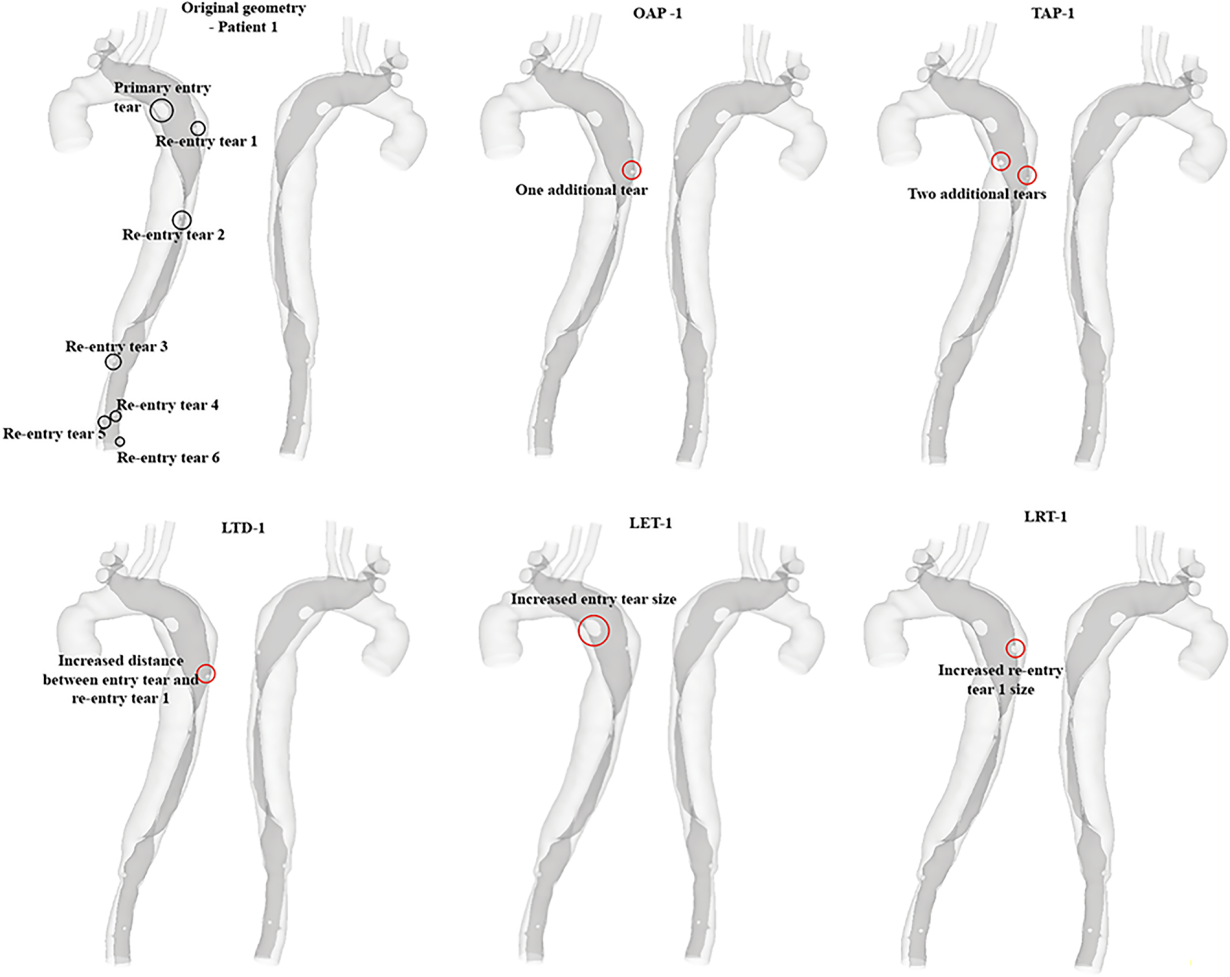
All reconstructed geometries and hypothetical models for patient 1 with regions of modifications being highlighted by red circles. OAP-1, creation of one proximal tear; TAP-1, creation of two proximal tears; LTD-1, increased tear distance between the entry and re-entry tear 1; LET-1, increased entry tear size; LRT-1, increased re-entry tear size.

**Figure 2:**
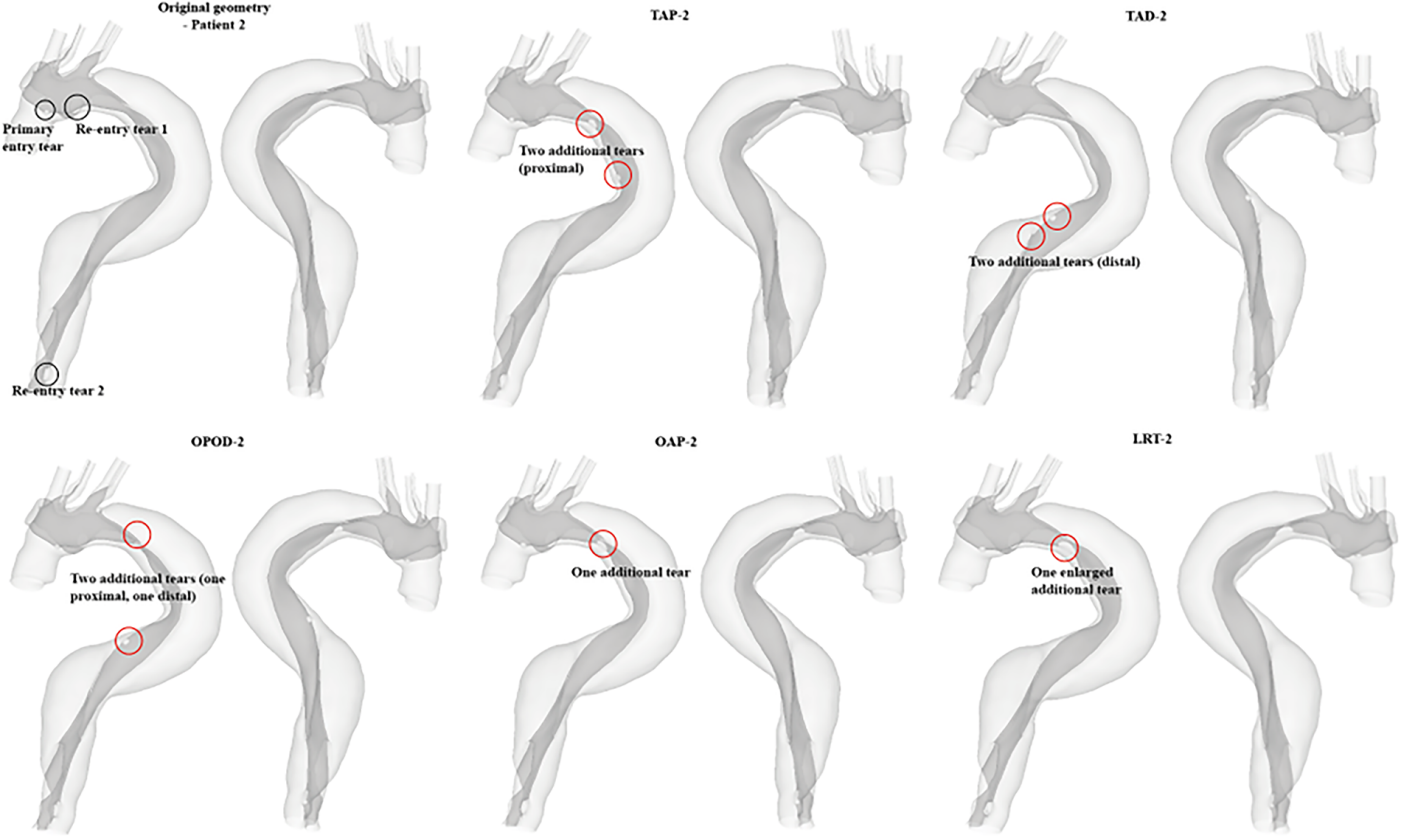
All reconstructed geometries and hypothetical models for patient 2 with regions of modifications being highlighted by red circles. TAP-2, creation of two proximal tears; TAD2, creation of two distal tears; OPOD-2, creation of one proximal tear and one distal tear; OAP-2, creation of one proximal tear; LRT-2, creation of one larger proximal tear.

The hypothetical models for each patient and their representative keys are summarized in Table 2 and presented in Figures 1, 2. For both patients, the effect of tear number was investigated by creating additional re-entry tears at the proximal DA. The effect of tear size was examined by increasing the entry tear and re-entry tear 1 in patient 1, and the additional re-entry tear (LRT-2) in patient 2. The sizes of additional re-entry tears and artificially enlarged tears were randomly chosen since it was difficult to control the actual size of a tear accurately. The effect of tear location was assessed by increasing the distance between the entry tear and re-entry tear 1 in patient 1, and by changing the location of two additional tears in patient 2.

**Table 2:** Summary of all modified geometries and the corresponding keys.

	Key	Modifications
Patient 1	OAP-1	One additional tear at the proximal DA (area: 13.6 mm^2^, distance to LSCA: 85.8 mm)
	TAP-1	Two additional tears at the proximal DA (areas: 15.1 mm^2^ and 4.4 mm^2^, distances to LSA: 70.6 mm and 85.8 mm)
	LTD-1	Increased distance between the entry and re-entry tear 1 (from 21.4 mm to 49.5 mm, area was also slightly increased from 10.6 mm^2^ to 11.5 mm^2^)
	LET-1	Increased entry tear size (area increased from 108.4 mm^2^ to 136.1 mm^2^)
	LRT-1	Increased re-entry tear size (area increased from 10.6 mm^2^ to 29.3 mm^2^)
Patient 2	TAP-2	Two additional tears at the proximal DA (areas: 45.4 mm^2^ and 16.4 mm^2^, distances to LSA: 51.2 mm and 115.4 mm)
	TAD-2	Two additional tears at the distal DA (areas: 19.9 mm^2^ and 41.8 mm^2^, distances to LSA: 174.3 mm and 215.4 mm)
	OPOD-2	Two additional tears: one at the proximal DA (area: 45.4 mm^2^, distance to LSA: 51.2 mm), while another one at the distal DA (area: 19.9 mm^2^, distance to LSA: 174.3 mm)
	OAP-2	One additional tear at the proximal DA (area: 45.4 mm^2^, distance to LSA: 51.2 mm)
	LRT-2	One additional tear at the proximal DA with increased tear size (area: 107.6 mm^2^, distance to LSA: 51.2 mm)

Patient1: OAP, one additional proximal tear; TAP, two additional proximal tears; LTD, larger tear distance; LET, larger entry tear; LRT, larger re-entry tear.

Patient2: TAP, two additional proximal tears; TAD, two additional distal tears; OPOD, one additional proximal tear and one additional distal tear; OAP, one additional proximal tear; LRT, larger additional re-entry tear.

All the reconstructed geometries were then imported into ANSYS ICEM CFD 19.2 (ANSYS, Canonsburg, PA, US) for mesh generation. Each computational mesh consisted of tetrahedral elements in the core and a minimum of 10 layers of prismatic cells in the fluid boundary layer. Local mesh refinement was performed in regions around the tears and great curvature. Grid independence tests were conducted, and an average element of 5.56 million for patient 1 and 7.02 million for patient 2 were adopted in the final analysis.

### 2.2. Computational fluid dynamics simulations

Details of the applied boundary conditions can be found in our previous study (19). In brief, an *in vivo* measured flow waveform was adopted from the literature (20), which was scaled based on patient-specific heart rate and maximum velocity measured by Doppler ultrasound. The scaled flow waveform was then imposed at the model inlet along with the assumption of a flat velocity profile. Regarding the outlet boundary condition, a 3-element Windkessel model (3-EWM) was applied at each outlet to describe the downstream vasculature. A reduction in compliance of the aortic wall is commonly observed in TAAD patients during the chronic phase (21), therefore, the aortic wall and intimal flap were assumed to be rigid and no-slip boundary conditions were applied.

Blood flow was assumed to be incompressible with a constant density of 1,060 kg/m^3^, which can be described by the following continuity (Equation 1) and momentum conservation equations (Equation 2).

(1) $$\begin{array}{c} \nabla u=0\; \end{array}$$

(2) $$\begin{array}{c} \frac{\partial (\rho u)}{\partial t}+\;\nabla \cdot (\rho uu)=-\nabla p+\nabla \cdot \tau +\;\rho f \end{array}$$

where u is the velocity vector, ρ is the blood density, ∇ is the divergency operator, p is the pressure, τ represents the viscous stress tensor, and **f** indicates the body force acting on the fluid per unit volume. Blood is a shear-thinning fluid, and its non-Newtonian behaviour was described using the empirical Carreau-Yasuda model (Equation 3):

(3) $$\begin{array}{c} \mu (\dot{\gamma })=\mu_{\infty }+(\mu_{0}-\mu_{\infty }){\left(1+{\left(\lambda \dot{\gamma }\right)}^{a}\right)}^{\frac{n-1}{a}}\; \end{array}$$

where, $\mu_{\infty }$ and $\mu_{0}$ are the infinite shear viscosity and the zero-shear viscosity with values being 0.0035 Pa s and 0.1600 Pa s, respectively, $\dot{\gamma }$ is the shear rate, and *a*, n, and λ are empirical constants with values being 0.64, 0.2128, and 8.2 s, respectively (22).

Flow simulations were carried out using ANSYS CFX 15.0 (ANSYS, Canonsburg, PA, US). A shear-stress transport transitional (SST-Tran) model (23) was applied to capture any possible turbulent flow. A high order advection scheme and second-order implicit backward Euler scheme were chosen for spatial and temporal discretisation, respectively. A fixed time-step of 0.001 s was used and the period of one cardiac cycle was 0.8 s based on the patient's heart rate (75 bpm in both patients). Moreover, a maximum root-mean-square residual of 1 × 10^–5^ was specified as a convergence criterion. All simulations were run for 4 cardiac cycles to reach a periodic stable solution. Results obtained from the 4th cycle were analysed using CEI Ensight 10.2 (CEI Inc, Apex, NC, US).

## 3. Results

### 3.1. Flow characteristics

**Figure 3:**
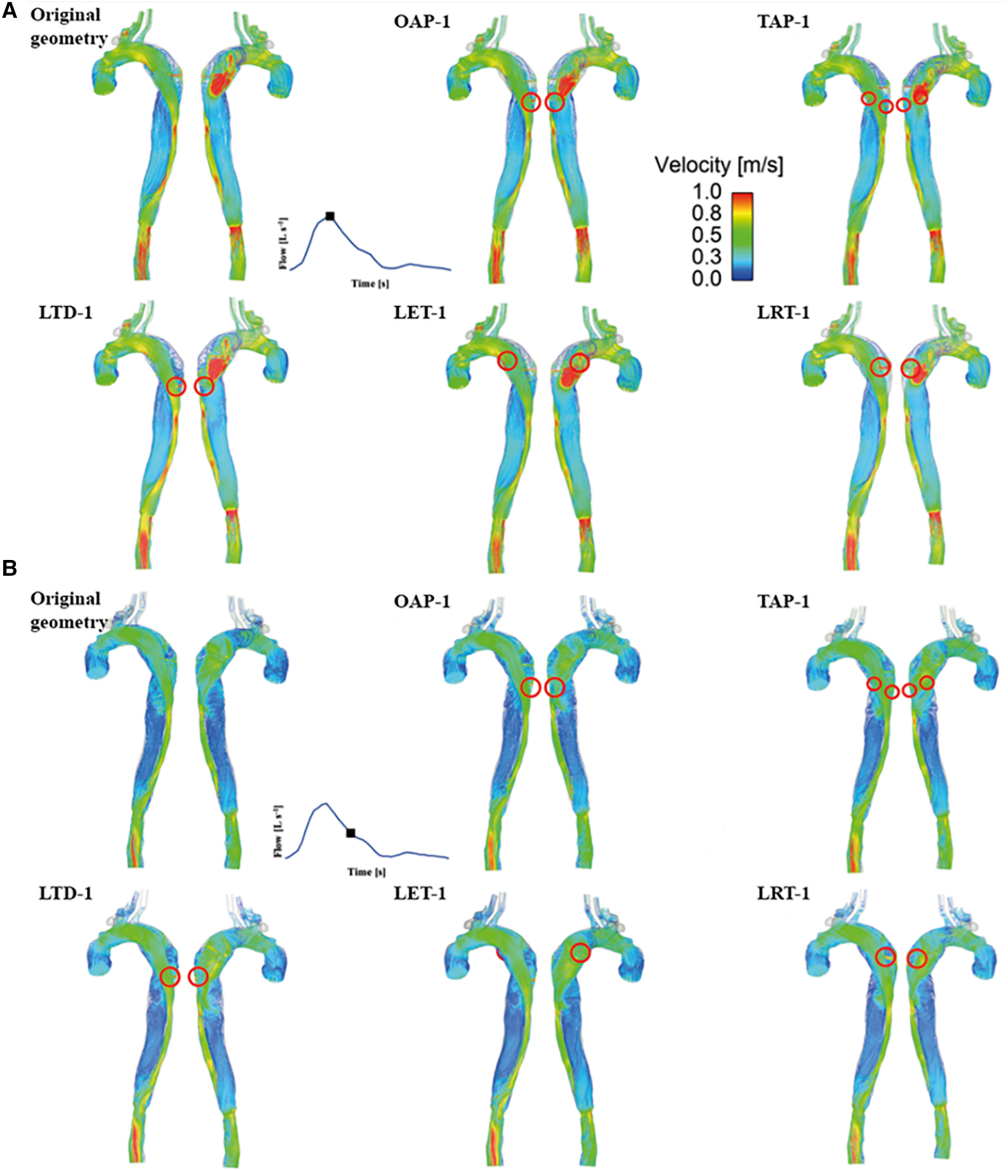
Velocity streamlines for patient 1 and the corresponding modified models at (**A**) peak systole and (**B)** mid-systolic deceleration. OAP-1, creation of one proximal tear; TAP-1, creation of two proximal tears; LTD-1, increased tear distance between the entry and re-entry tear 1; LET-1, increased entry tear size; LRT-1, increased re-entry tear size.

**Figure 4:**
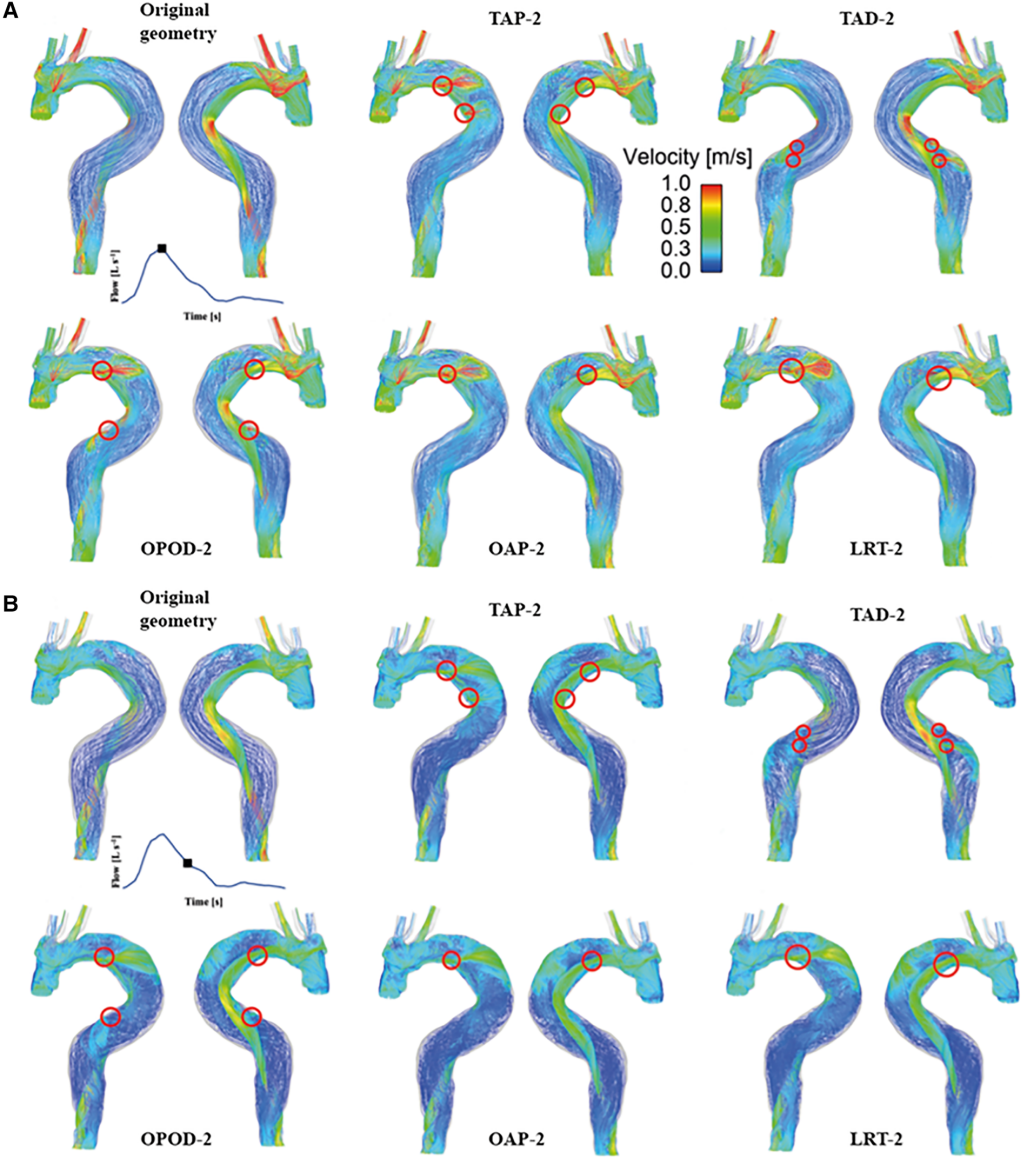
Velocity streamlines for patient 2 and the corresponding modified models at (**A**) peak systole and (**B**) mid-systolic deceleration. TAP-2, creation of two proximal tears; TAD2, creation of two distal tears; OPOD-2, creation of one proximal tear and one distal tear; OAP-2, creation of one proximal tear; LRT-2, creation of one larger proximal tear.

Instantaneous velocity streamlines for all models were captured at two systolic time points, namely, peak and mid systole to present flow patterns (Figures 3, 4). Although there were minor differences in the modified regions (as indicated by the red circles), the overall flow patterns were comparable with obviously higher velocities being observed in the TL than FL due to the compressed lumen areas. However, patient 1 showed higher FL velocities as compared to patient 2, which might result from the presence of a larger number of re-entry tears. Moreover, regions with reduced lumen areas led to blood flow acceleration, forming localized high velocity flow jets. At the mid-systolic deceleration, flow patterns became more complex with helical flow, flow separation and recirculation.

Quantitative results of mean (V_mean_) and maximum (V_max_) cycle-maximum velocity were compared (Table 3), where cycle-maximum velocity is defined as the maximum velocity recorded at a given time point in the cardiac cycle. Increasing either the tear size or number of re-entry tears caused a decrease in V_mean_ and V_max_, whereas increasing tear distance (LTD-1, and OAOP-2 compared with TAP-2 or TAD-2) had negligible effect on blood velocities.

**Table 3:** Summary of the mean cycle-maximum velocity, and maximum cycle-maximum velocity for all geometries.

Patient	Geometry	V_mean_[m s^−1^]	Δ	V_max_ [m s^−1^]	Δ
1	Patient-1	0.82	–	1.72	–
	OAP-1	0.79	−3.7%	1.66	−3.5%
	TAP-1	0.77	−6.1%	1.61	−6.4%
	LTD-1	0.82	0.0%	1.73	0.6%
	LET-1	0.77	−6.1%	1.59	−7.6%
	LRT-1	0.77	−6.1%	1.59	−7.6%
2	Patient-2	0.86	–	1.89	–
	TAP-2	0.73	−15.0%	1.61	−14.8%
	TAD-2	0.76	−11.6%	1.65	−12.7%
	OPOD-2	0.74	−14.0%	1.66	−12.2%
	OAP-2	0.71	−17.4%	1.61	−14.8%
	LRT-2	0.68	−20.9%	1.60	−15.3%

Patient1: OAP, one additional proximal tear; TAP, two additional proximal tears; LTD, larger tear distance; LET, larger entry tear; LRT, larger re-entry tear.

Patient2: TAP, two additional proximal tears; TAD, two additional distal tears; OPOD, one additional proximal tear and one additional distal tear; OAP, one additional proximal tear; LRT, larger additional re-entry tear.

### 3.2. Time averaged wall shear stress (TAWSS)

**Figure 5:**
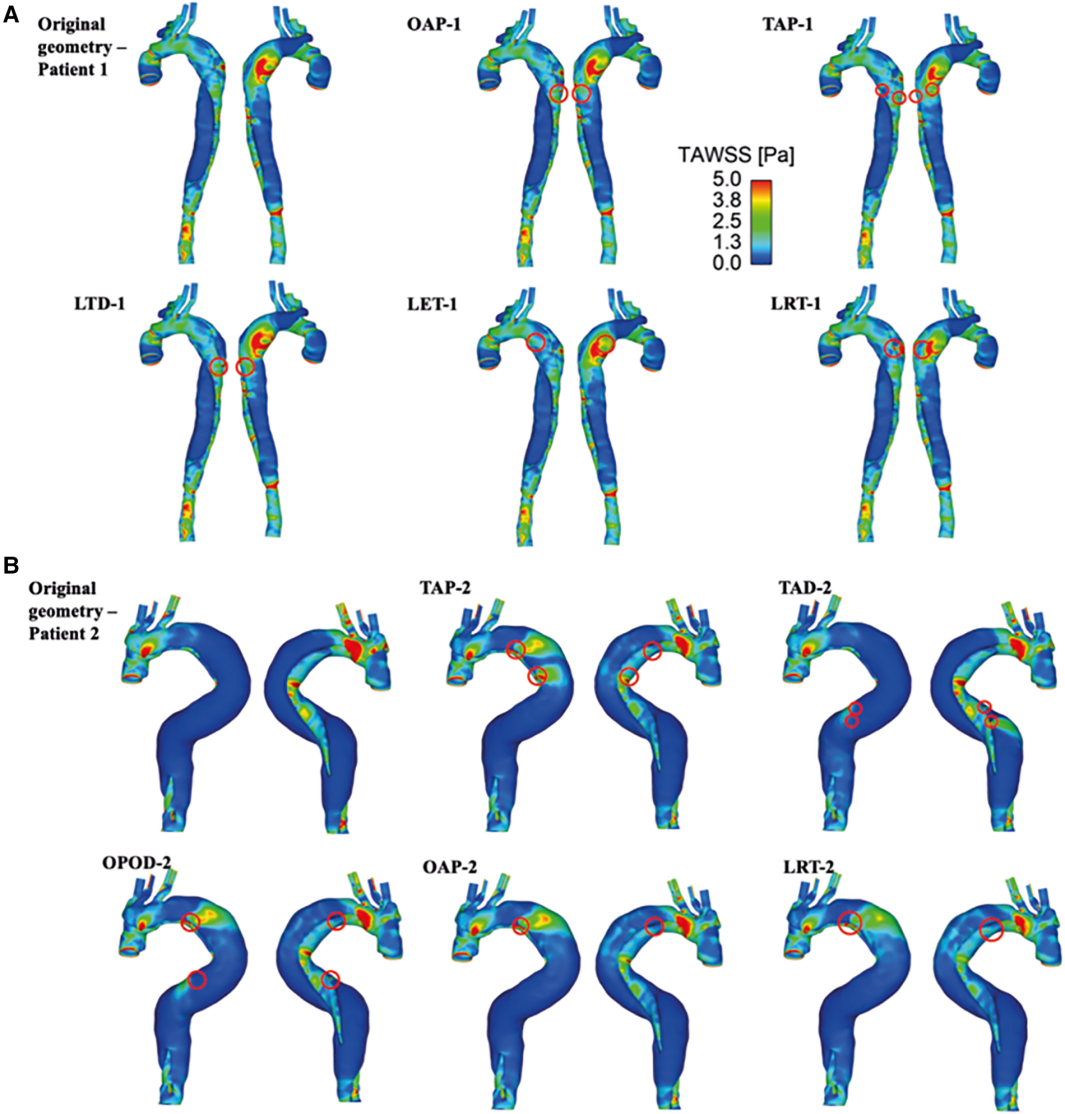
TAWSS for (**A**) patient 1 and the corresponding modified models: OAP-1, creation of one proximal tear; TAP-1, creation of two proximal tears; LTD-1, increased tear distance between the entry and re-entry tear; LET-1, increased entry tear size; LRT-1, increased re-entry tear size, and (**B**) patient 2 and the corresponding modified models: TAP-2, creation of two proximal tears; TAD-2, creation of two distal tears; OPOD-2, creation of one proximal tear and one distal tear; OAP-2, creation of one proximal tear; LRT-2, creation of one larger proximal tear.

TAWSS contours are shown for all the simulated models (Figure 5). For both patients, High TAWSS (> 5 Pa, shown in red) were observed on the FL walls opposite the entry and re-entry tears. This can be explained by that blood flow was accelerated when passing through the tears, and the high velocity jet impinged directly on the opposing aortic wall causing high WSS. High TAWSS values were also observed in regions surrounding the tears and in the distal regions with reduced lumen areas, coinciding with the regions experiencing blood flow acceleration (Figures 3, 4).

**Figure 6:**
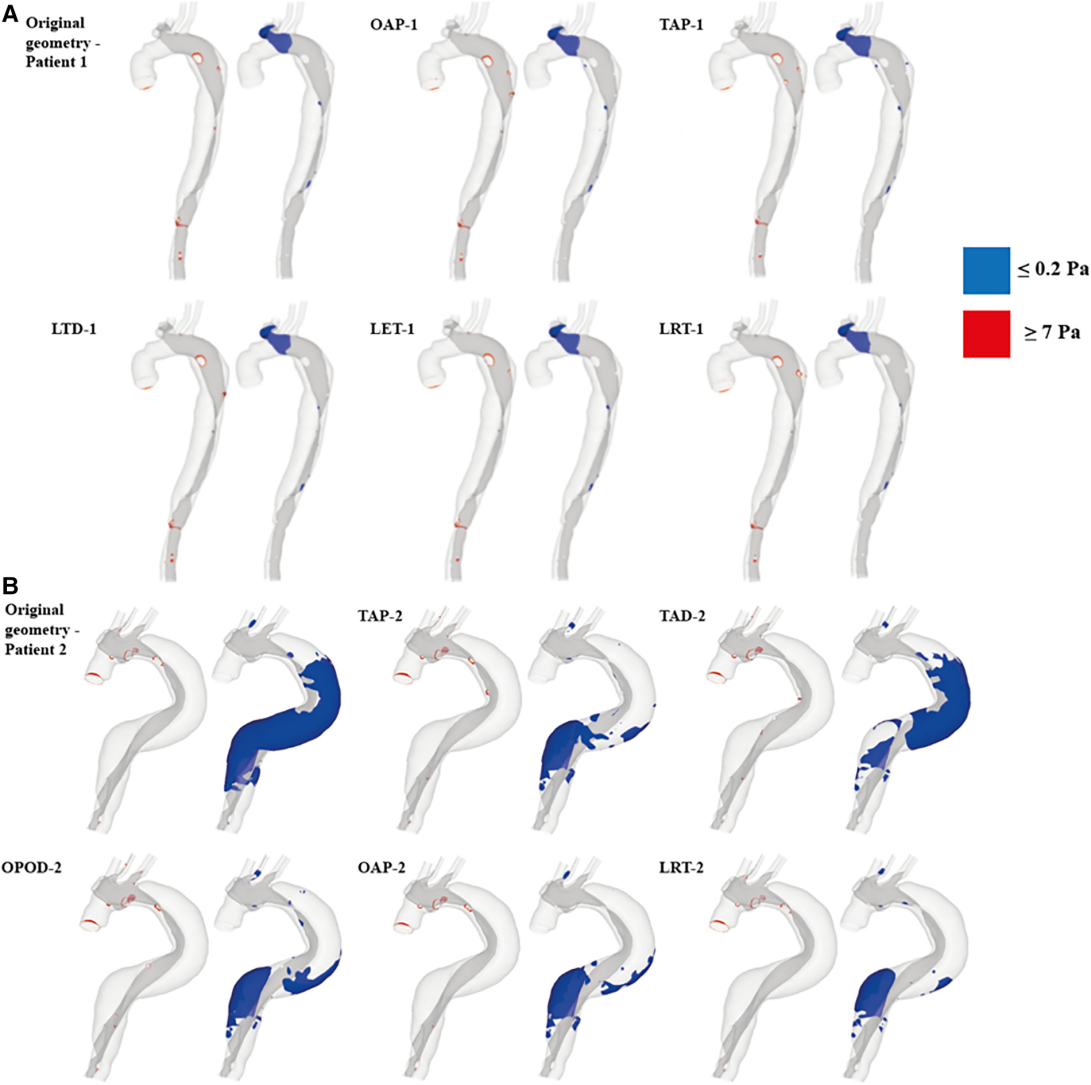
TAWSS iso-surfaces for (**A**) patient 1 and (**B**) patient 2 are displayed for regions with high (≥7 Pa, red) and low (≤0.2 Pa, blue) values. OAP-1, creation of one proximal tear; TAP-1, creation of two proximal tears; LTD-1, increased tear distance between the entry and re-entry tear; LET-1, increased entry tear size; LRT-1, increased re-entry tear size; TAP-2, creation of two proximal tears; TAD-2, creation of two distal tears; OPOD-2, creation of one proximal tear and one distal tear; OAP-2, creation of one proximal tear; LRT-2, creation of one larger proximal tear.

In models OAP-1 (creation of one additional tear), TAP-1 (creation of two additional tears), and LRT-1 (larger re-entry tear), there were small reductions in the area exposed to high TAWSS surrounding the tears (as highlighted by red circles). On the contrary, additional tears in patient 2 caused high TAWSS concentrations on the FL walls, which were also highlighted. Moreover, iso-surfaces of TAWSS values greater than 7 Pa (shown in red) and less than 0.2 Pa (shown in blue) were calculated and displayed in Figure 6. Similar to TAWSS distributions (Figure 5), high TAWSS concentrations were located in regions surrounding the tears, whereas extremely low TAWSS values appeared mainly in the proximal FL of patient 1, and along the entire FL of patient 2. In both patients, changing tear configurations had minor impacts on regions with high TAWSS, but significantly reduced the low TAWSS regions in patient 2.

Table 4 summarizes the maximum TAWSS, together with areas of the regions with high (≥7 Pa) and low (≤0.2 Pa) TAWSS values in all the simulated models. The peak TAWSS values were greatly reduced by increasing the number or size of tears, more apparently for patient 1, where the maximum TAWSS was reduced by 40.9% by creating an addition tear (OAP-1). Increasing the size of either entry (LET-1) or re-entry tear (LRT-1) had even more impacts on the predicted peak TAWSS by decreasing the value of up to 44%. However, areas exposed to either high or low TAWSS were comparable among all the simulated models of patient 1. Regarding patient 2, the most obvious reduction in the peak TAWSS value (reduced by 28%) was observed in the model with a single additional tear (OAP-2). Increasing the size of this additional tear (LRT-2) resulted in the largest area reductions in both high and low TAWSS regions, by approximately 66% and 72% respectively.

**Table 4:** Maximum TAWSS, areas of the regions with high (≥7 Pa) and low (≤0.2 Pa) TAWSS values recorded for all models.

Patient	Geometry	TAWSS [Pa]	Area (cm^2^) TAWSS ≥7 Pa	Area (cm^2^) TAWSS ≤0.2 Pa
1	Patient-1	47.2	28.7	286.6
	OAP-1	27.9	28.5	287.2
	TAP-1	26.7	28.4	300.3
	LTD-1	30.7	33.0	266.6
	LET-1	26.4	26.0	280.1
	LRT-1	26.4	28.2	278.4
2	Patient-2	31.8	63.2	2413.0
	TAP-2	25.3	28.3	906.5
	TAD-2	27.6	29.9	1470.6
	OPOD-2	24.6	30.6	1003.0
	OAP-2	22.9	25.3	896.4
	LRT-2	26.3	21.6	675.1

Patient1: OAP, one additional proximal tear; TAP, two additional proximal tears; LTD, larger tear distance; LET, larger entry tear; LRT, larger re-entry tear.

Patient2: TAP, two additional proximal tears; TAD, two additional distal tears; OPOD, one additional proximal tear and one additional distal tear; OAP, one additional proximal tear; LRT, larger additional re-entry tear.

### 3.3. Luminal pressure difference (LPD)

**Figure 7:**
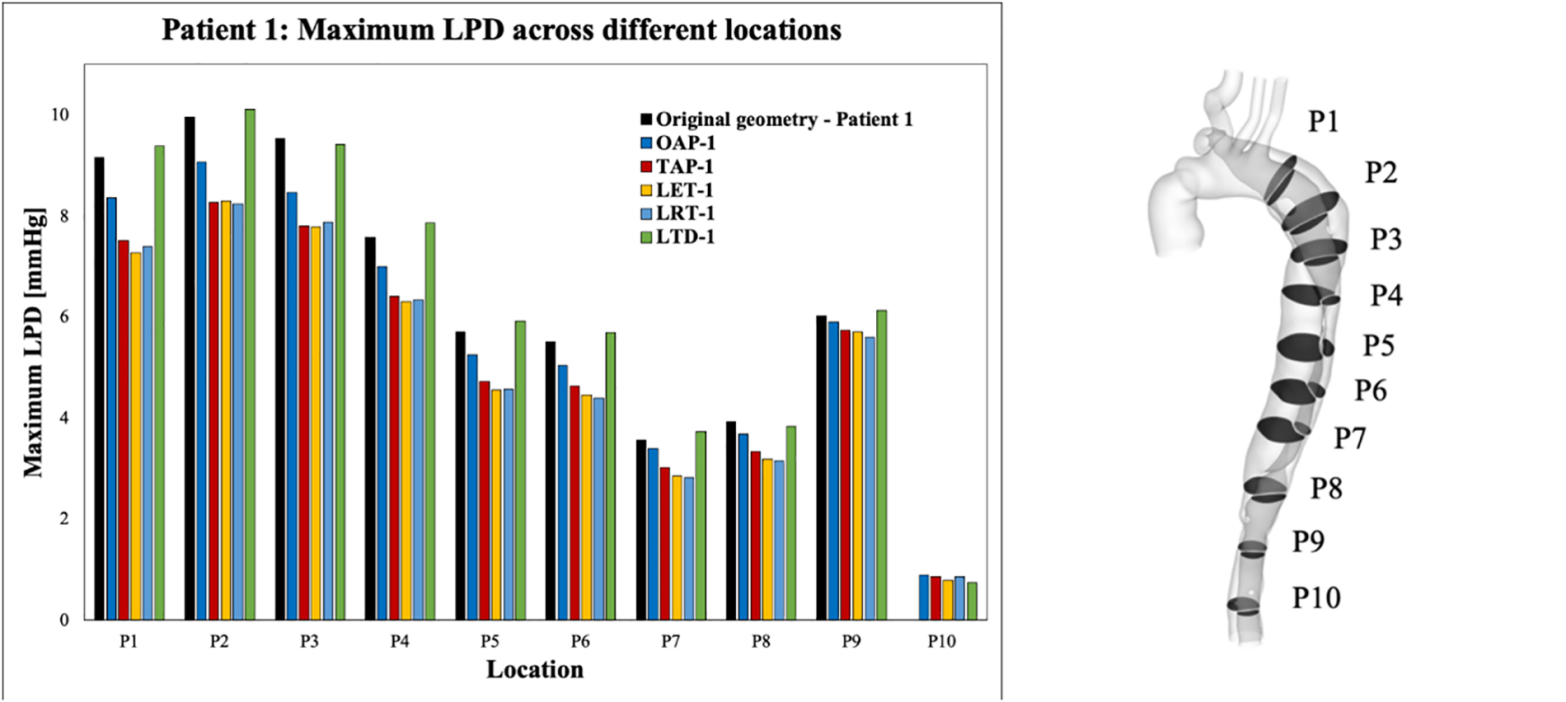
Summary of LPD along the aorta for patient 1 and the corresponding modified geometries: OAP-1, creation of one proximal tear; TAP-1, creation of two proximal tears; LTD-1, increased tear distance between the entry and re-entry tear; LET-1, increased entry tear size; LRT-1, increased re-entry tear size.

**Figure 8:**
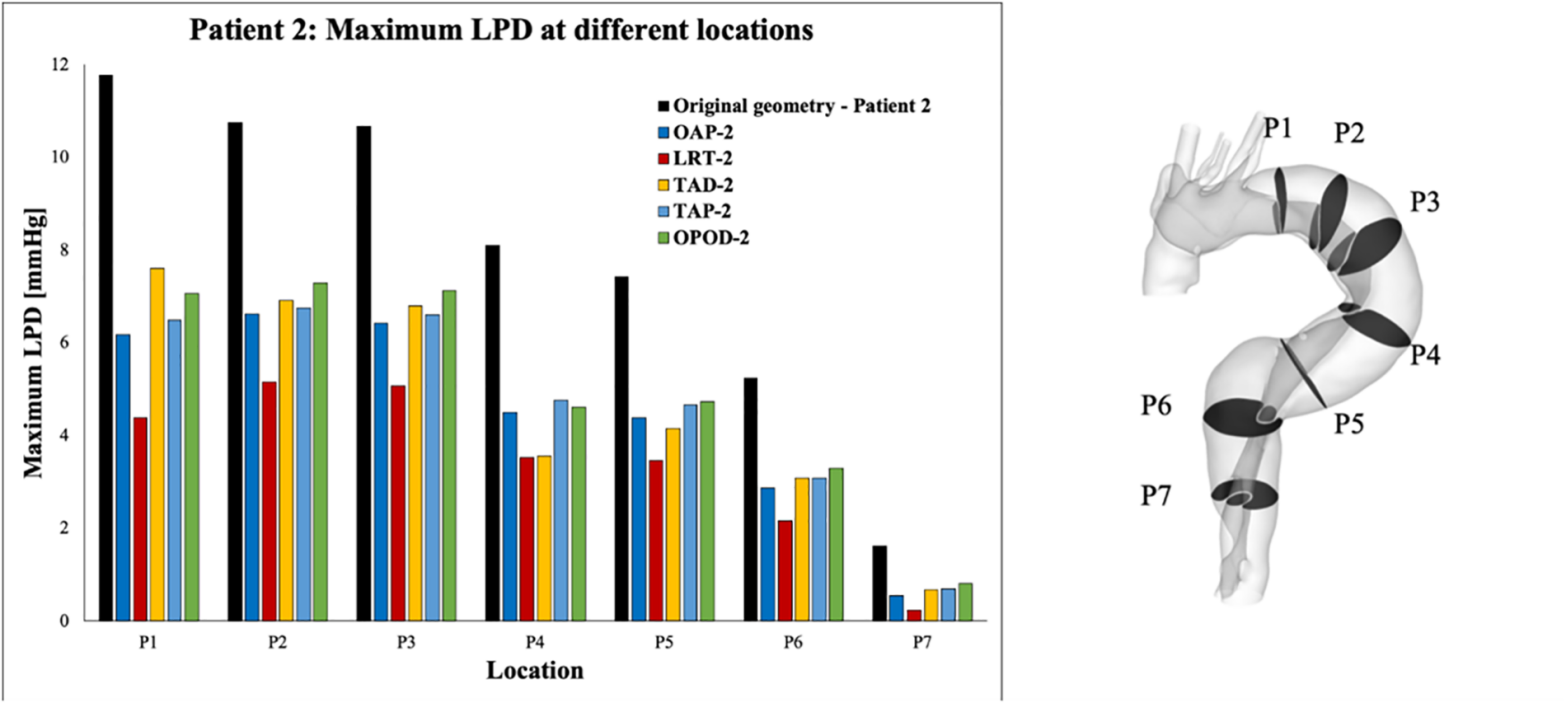
Summary of LPD along the aorta for patient 2 and the corresponding modified geometries: TAP-2, creation of two proximal tears; TAD-2, creation of two distal tears; OPOD-2, creation of one proximal tear and one distal tear; OAP-2, creation of one proximal tear; LRT-2, creation of one larger proximal tear.

For each simulated model, pressures were evaluated at ten cross-sectional planes with equal distances along the aorta. Among each cross-sectional plane, LPD was calculated as the difference of space-averaged true and false lumen pressures ($LPD=P_{TL}-P_{FL}$). The maximum LPD over a cardiac cycle for each plane was then determined for all the simulated models and plotted as a bar chart (Figures 7, 8).

Across all the selected planes, the maximum LPD was observed at plane 2 (P2), located distally to the entry-tear of patient 1, and at P1 for patient 2. Therefore, P2 and P1 will be used as representative planes for patients 1 and 2, respectively, to calculate the variations in mean and maximum LPD when compared with the relevant original cases (Table 5).

**Table 5:** Maximum and mean LPD reduction for all modified geometries.

Patient	Geometry	Mean LPD Reduced [mmHg]	Max. LPD Reduced [mmHg]
1	OAP-1	0.53	1.08
	TAP-1	1.12	1.74
	LTD-1	−0.13	0.12
	LET-1	1.17	1.76
	LRT-1	1.17	1.88
2	TAP-2	3.22	5.28
	TAD-2	3.26	4.55
	OPOD-2	2.95	4.70
	OAP-1	3.44	5.58
	LRT-2	4.51	7.39

Patient1: OAP, one additional proximal tear; TAP, two additional proximal tears; LTD, larger tear distance; LET, larger entry tear; LRT, larger re-entry tear.

Patient2: TAP, two additional proximal tears; TAD, two additional distal tears; OPOD, one additional proximal tear and one additional distal tear; OAP, one additional proximal tear; LRT, larger additional re-entry tear.

Overall, both the mean and maximum LPD reduced in all the modified geometries, except for the model with an increased distance between the entry tear and re-entry tear (LTD-1), where the mean LPD was increased by 0.13 mmHg. For patient 1, the mean LPD values were decreased by 6.6% (0.53 mmHg) and 13.6% (1.12 mmHg) for the models with one (OAP-1) and two (TAP-1) additional tear(s), respectively. Regarding patient 2, creating additional tears resulted in much greater reductions in both mean and maximum LPD values, ranging from 3.22 to 4.51 mmHg, and 4.55 to 7.39 mmHg, respectively.

In addition, comparison of OAP-2 (creation of one additional tear) and LRT-2 (creation of one larger additional tear) allowed analysis of the influence of increasing tear size on LPD. The mean LPD in LRT-2 was decreased by 60.2% (4.51 mmHg), as compared to 46.1% (3.44 mmHg) in OAP-2, suggesting a positive correlation between the tear size and the LPD reduction. This was further proved by patient 1, in which the mean LPD was decreased by 1.17 mmHg with an increased entry (LET-1) or re-entry tear (LRT-1) size. In fact, for both patients, enlarging the tear size had more prominent impact on LPD than increasing tear numbers.

Furthermore, comparison of TAP-2, TAD-2, and OPOD-2 allowed assessment of the influence of tear locations. Having two additional tears in either the proximal DA (TAP-2) or the distal DA (TAD-2) achieved greater reduction in mean LPD (3.22 mmHg, 42.5% and 3.26 mmHg, 43.8% respectively) in comparison to the model with one tear in the proximal DA and another in the distal (OPOD-2, 38.9%, 2.95 mmHg). Increasing the tear distance in patient 1 (LTD-1) showed less effect on LPD, while reducing the distance between the entry and re-entry tear in patient 2 resulted in larger reduction in maximum LPD: model TAP-2 showed a maximum LPD reduction of 5.28 mmHg in comparison with TAD-2 (4.55 mmHg) and OPOD-2 (4.70 mmHg).

## 4. Discussion

In an effort to understand the underlying mechanisms for aneurysmal dilatation in surgically repaired TAAD patients, and to elucidate the role of anatomical factors in dissection progression, the influence of tear size, location, and number on haemodynamic parameters was examined through CFD simulations. This involved reconstruction of two patient-specific geometries from CTA images, artificial modification of these geometries to obtain a total of five hypothetical models per patient, and the setting of appropriate boundary conditions. Comparisons of the modified geometries allowed direct analysis of the changes in, if any, haemodynamic outcomes.

Regions with chronic exposure to elevated WSS conditions have been associated with increased risk of local aortic wall degradation, and late vessel enlargement (24, 25). The maximum TAWSS was greatly reduced in all the hypothetical models with additional tears, indicating the potential role of increased tear numbers in preventing vessel wall degradation. Similar finding of reduced WSS surrounding the tears as a result of creation of additional tears has been reported in a previous study using a TBAD swine model (10). The reduced maximum TAWSS magnitudes might result from the decreased velocity magnitudes, as reported in Table 3. In patient 1, the maximum velocity (V_max_) was reduced by 3.5% with one additional re-entry tear (OAP-1), and was further decreased by 6.4% by creating one more re-entry tear (TAP-1). In patient 2 where the entry tear size was much smaller than in patient 1, introducing additional re-entry tears had a more pronounced effect on reducing the V_max_, ranging from 12.2% to 14.8% among the models.

Recent studies on repaired TAAD have identified an association between elevated LPD and rapid aortic dilatation (19, 26). Furthermore, patients with unstable aortic growth were found to have fewer number of re-entry tears, although the correlation between LPD and number of re-entry tears was not examined for each individual patient (19). The influence of the number of re-entry tears on LPD has also been of emerging interest, which has been extensively studied in TBAD (9–11, 13–15). It was suggested the re-entry tears could dampen the TL/FL pressure difference by allowing inter luminal flow exchange (9–11, 13, 14, 27). Armour et al. (10) among the others, found that both FL pressure and LPD were significantly reduced by increasing the number of re-entry tears. Analysis of both patients in this study showed a positive correlation between the number of re-entry tears and LPD reduction, in consistency with the observations reported in the literature (Figures 7, 8).

Except for the number of re-entry tears, this study also demonstrated the influence of tear size on LPD. In fact, for both patients, enlarging the entry and re-entry tears had a more marked effect on reducing LPD than introducing additional tears. For example, for patient 1, enlarged entry tear (LET-1) and enlarged re-entry tear (LRT-1) both reduced mean LPD by 1.17 mmHg, obviously higher than the cases with one or two additional tear(s) (OAP-1: 0.53 mmHg and TAP-1: 1.12 mmHg). This might be associated with the increase in total tear areas, such that area was increased by 27.7 mm^2^ with model LET-1, compared to 13.6 mm^2^ and 19.5 mm^2^, respectively for models OAP-1 and TAP-1. However, in model LRT-1, the tear area was increased by 18.7 mm^2^, lower than that of model TAP-1, but resulted in higher LPD reduction, suggesting that enlarging a single proximally located re-entry tear in the descending aorta would be more effective at reducing LPD than creating two distal tears even with slightly larger areas. This was further supported by results obtained for patient 2, where LRT-2 with increased re-entry tear size reduced mean LPD by 4.51 mmHg which was significantly higher than all the other models with two additional tears (TAP-2, TAD-2, and OPOD-2) (Table 5).

The maximum TAWSS (Table 4) and cycle-maximum velocity (Table 3) were also reduced by increasing the size of tears in both patients. The results agreed well with previous *in vitro* and *ex vivo* studies, where smaller tear size was correlated with greater LPD, WSS, and tear velocity (9, 28). However, as aforementioned, increasing entry tear size may cause greater FL flow (16), and therefore higher risk of aneurysmal expansion in TBAD (17).

The normal range of WSS was reported to be 1–7 Pa (29), and hence regions with TAWSS higher than 7 Pa were identified and compared (Figure 6 and Table 4). In patient 1, all the hypothetically created models had slightly smaller areas of high TAWSS as compared to the original model, except for the model LTD-1 with an increased distance between the entry tear and re-entry tear, which caused 16.7% increase in high TAWSS area. Regarding patient 2, the surface areas exposed to high TAWSS were greatly reduced in all the hypothetical models, with reductions ranging from 52% - 72%. Adding tears in the proximal region (OAP-2, TAP-2, and LRT-2) led to higher area reductions than the models with either two distal tears (TAD-2) or one proximal and one distal tear (OPOD-2). On the other hand, regions with low TAWSS (≤0.2 Pa) were also compared since shear stresses lower than 0.2 Pa have been reported to promote thrombus formation (30). The areas of low TAWSS regions were markedly reduced in patient 2 by creating additional tears, and again, more obvious reductions were found in models with one (OAP-2 and LRT-2) or two (TAP-2) proximal re-entry tears. Comparable low TAWSS areas were observed among all the models of patient 1.

The location of re-entry tears is also of great interest, and our computational results showed that creating a re-entry tear closer to the entry tear had a greater impact on the local haemodynamics than adding a distal re-entry tear. This was evidenced by the fact that the least reduction in peak TAWSS and LPD values were achieved with models LTD-1 and TAD-2 which had larger distances between the entry and re-entry tears in patients 1 and 2, respectively. Moreover, greater reductions in mean LPD were obtained with models TAP-2 (two additional proximal tears) and TAD-2 (two additional distal tears) than with model OAOP-2 (two additional tears, one proximal and one distal), suggesting that larger mean LPD reduction might be caused by two adjacent tears.

Elevated LPD (>5 mmHg) has been potentially associated with progressive aortic dilatation (26). A recent numerical study has also shown that at least 50% reduction in the pressure between the true and false channel is needed to stabilize a dissection (31). In both patients examined here, the overall best performance was achieved by increasing the size of re-entry tear (LRT-1 and LRT-2), especially for patient 2, the maximum LPD was reduced to 4.4 mmHg from 11.8 mmHg (62.7% reduction). Nevertheless, LPD reduction was greater in patient 2 than patient 1. This is likely to be due to the anatomical differences between the two patients. Patient 1 presented with a large entry tear and 6 small re-entry tears, whereas patient 2 had a small entry tear and two large re-entry tears which were 354 mm apart. As expected, adding tears between the two distant re-entry tears in patient 2 resulted in greater LPD reduction.

Patients with a patent FL following surgical ascending replacement of TAAD have been associated with progressive aortic dilatation (32–34). Reoperation by either surgical resection or stent-graft coverage of one or all the remaining tears is required to manage and prevent aortic rupture, despite the high risks and costs associated with the procedures. In this study, through CFD simulations of different hypothetical models modified on the basis of two patient-specific repaired TAAD geometries, we found that increasing the number or size of re-entry tears reduced LPD and maximum TAWSS, which may help stabilize aortic growth (19, 26). Although these findings were based on hypothetical scenarios, they could provide theoretical ground for the use of fenestration as a potential alternative in managing unstable aortic growth.

The current study has several limitations. First, computational models with various tear configurations were built based on two patient-specific geometries. The impact of entry or re-entry tears on local haemodynamics may vary with anatomical features of AD so further validation of the current findings in a large patient cohort will be required. Second, the rigid wall assumption was reported to underestimate the true and false lumen pressures (35) as well as to predict inaccurate low WSS values and distributions (36, 37), but it has been found to have a minor effect on the predicted LPD and maximum TAWSS values (37). Since LPD is the main parameter of interest here and considering the 8–10 fold increase in computational time, fluid-structure interaction simulations were not deemed necessary and practical for the purpose of the present study. Third, non-patient-specific inlet flow waveforms were used in the CFD simulations. Although this assumption can have a significant influence on the predicted flow and pressure in TBAD (38, 39), it is not expected to affect the findings reported in this study. Finally, there was no direct validation of the computational results as 4D-flow magnetic resonance imaging data of the two patients were not available. Nevertheless, the computational methodology adopted in the present study has been validated in previous TBAD studies, showing a good overall agreement with available *in vivo* measurements (20, 40).

## 5. Conclusion

This study comprehensively examined the role of tears in haemodynamics in surgically repaired TAAD patients, with a particular focus on LPD. Increasing the number of re-entry tears and size of both entry and re-entry tears decreased LPD, peak TAWSS values, as well as regions with abnormally high or low TAWSS magnitudes, thereby potentially slowing down aortic expansion. Larger tear size was found to be more influential on LPD reduction than including additional tears. Moreover, additional re-entry tears in the proximal descending aorta were more effective in reducing LPD than distal additional re-entry tears. These findings are promising and point out the potential role of creating a large-sized proximal fenestration in managing progressive aortic dilatation in repaired TAAD patients. Nevertheless, future studies with a large cohort of patients are warranted.

## Data Availability

The original contributions presented in the study are included in the article, further inquiries can be directed to the corresponding author.
